# Sensitivity Improvement of MEMS Resonant Accelerometers by Shape Optimization of Microlevers and Resonators

**DOI:** 10.3390/s25216807

**Published:** 2025-11-06

**Authors:** Longqi Ran, Wensheng Zhao, Ting Li, Jiangbo He, Wu Zhou

**Affiliations:** 1 School of Mechanical and Electrical Engineering, University of Electronic Science and Technology of China, Chengdu 611731, China; ranlongqi7@163.com (L.R.); li_ting25@163.com (T.L.); 2 School of Mechanical Engineering, Xihua University, Chengdu 610039, China; zhaowensheng99@stu.xhu.edu.cn

**Keywords:** MEMS, resonant accelerometers, shape optimization, sensitivity improvement

## Abstract

High-frequency sensitivity to external acceleration is crucial for improving the accuracy of MEMS resonant accelerometers. This study proposes utilizing shape optimization of microlevers and resonators to improve sensitivity. Initially, an optimization model for microlevers is established, considering the arm’s shape and the dimensions of the pivots, outputs, inputs, and supported beams. Secondly, shape optimization for the resonant beam of the tuning fork resonators is implemented, utilizing a bi-objective function to maintain the fundamental frequency. Finally, the genetic algorithm is employed in both optimizations to search for the global optimal solution. The microlever optimization achieves a high sensitivity of 286.9 Hz/g, and the final trapezoidal arm shape offers the advantage of accommodating a larger proof mass within a given die area. Meanwhile, the resonator optimization improves the sensitivity to axial inertial force from 727 Hz/mN to 1338.5 Hz/mN while keeping the fundamental frequency at approximately 20,000 Hz. Integrating the optimized microlevers and resonators yields a very high sensitivity of 480.2 Hz/g, and the sensitivity per proof mass area is significantly higher than that reported in previous studies.

## 1. Introduction

High-precision resonant accelerometers based on microelectromechanical system (MEMS) technology have broad applications, including in seismometers [1], inertial navigation [2], vibration detection [3], and inclination measurement [4]. Compared with capacitive techniques, resonant techniques enable designs that achieve higher sensitivity, better bias stability, and superior low-frequency noise performance without sacrificing mechanical robustness [5].

High-frequency sensitivity to external acceleration is essential for achieving high accuracy in MEMS resonant accelerometers. To date, methods such as novel resonator designs and microlevers have been employed to enhance sensitivity.

Ding et al. designed a rectangular resonator with a fishbone structure to enhance sensitivity [6]. Zhao et al. proposed a rectangular resonator using the second lateral transverse mode to achieve higher sensitivity [7]. Li et al. presented a high-resolution resonant accelerometer with an enhanced scale factor using a thermal boost approach to increase sensitivity, which features resonators consisting of a dual-clamped beam with two symmetrically arranged piezoresistive gauges. An electrostatic stiffness tuning resonator was also introduced to improve sensitivity [8,9]. However, the electrostatic stiffness tuning is more complex than mechanical stiffness tuning and needs a very high voltage (30–50 V) to generate electrostatic stiffness [10]. A high voltage is difficult to generate using an application-specific integrated circuit (ASIC) and introduces the risk of pull-in and even electrostatic breakdown. Thus, the mechanical stiffness tuning is more reliable.

The compliant microlever is another common method for improving sensitivity. In MEMS resonant accelerometers, the inertial force from external acceleration is applied axially to the resonant beam, shifting the natural frequency of the resonators. Microlevers enhance sensitivity by amplifying this inertial force.

The pivot’s position and the dimensions of microlevers can both affect the amplification factor. Su et al. established an analytical model to study the influence of the position and dimensions of the pivot and output beams on the amplification factor [11,12]. Comi et al. [13], Zou et al. [4], and Jia et al. [14] found that there is always a specific pivot position that maximizes the amplification factor. A new analytical model proposed by Ding et al. revealed that the pivot’s position and the dimensions of the pivot, the output beam, and the input beam all can affect the amplification factor [15]. Huang et al. analyzed the coupling effect of microlevers on the proof mass and integrated the resonant frequency of the proof mass, the natural frequency of the resonator, and the sensitivity of the accelerometer into a single model for structural cooperative design [16]. Fang et al. studied the enhancement of sensitivity through stiffness matching among the resonators, microlevers, and supported beams [17]. Zhang et al. applied the energy-consuming concept and the Nelder–Mead algorithm to address the design issues of microlevers and further increase the sensitivity of MEMS resonant accelerometers [18]. Meanwhile, several studies have proposed using multistage microlevers to achieve higher amplification factors, thereby enhancing the sensitivity of MEMS resonant accelerometers [19,20]. However, multistage microlevers may suffer from the attenuation of the effective amplification factor and an increase in structural complexity [21].

Thus far, studies on sensitivity improvement for MEMS resonant accelerometers have primarily focused on rectangular-shaped microlevers and resonators. This paper proposes implementing shape optimization of microlevers and resonators to improve sensitivity. Section 2 describes the working principle of MEMS resonant accelerometers. Section 3 and Section 4 present the optimization models for microlevers and resonators, respectively. Section 5 discusses the optimization results.

## 2. Principle and Structural Feature

**Figure 1:**
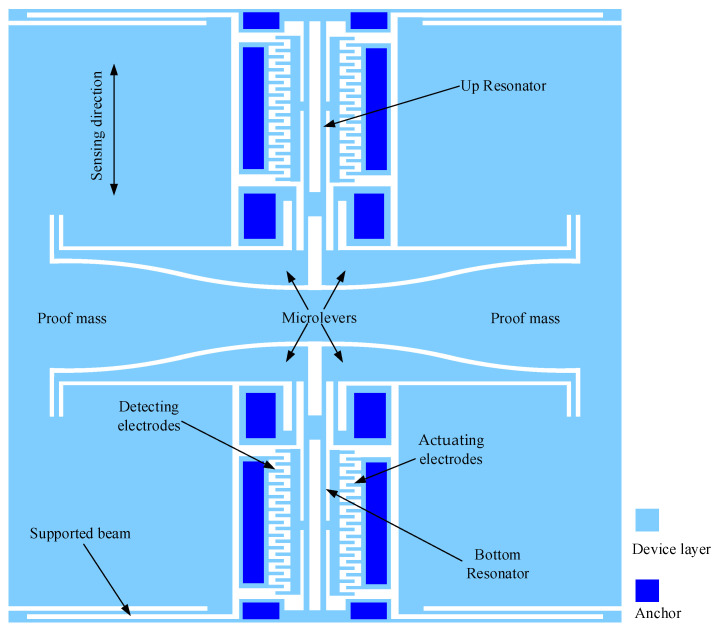
The schematic diagram of MEMS resonant accelerometers. A pair of tuning fork resonators is designed vertically to realize the differential sensing. Four supported beams bending vertically guarantee that the proof mass can only move vertically. Four microlevers are employed to amplify the inertial force generated by the external acceleration.

The MEMS resonant accelerometer studied in this paper comprises a proof mass, microlevers, supported beams, resonators, actuating electrodes, and detection electrodes, as shown in Figure 1. When an external acceleration is applied, the proof mass generates an inertial force. This force is amplified by the microlevers and then applied to the resonators as an axial load, causing the natural frequencies of the resonators to change—one resonator experiences a frequency increase while the other experiences a decrease. A pair of tuning fork resonators is employed for differential sensing, making the accelerometer insensitive to common-mode factors such as temperature-induced frequency variations. The tuning fork resonators operate in opposite modes to isolate them from the effects of the microlevers, except for the transmission of axial loads. Electrostatic comb electrodes are used to suppress resonant nonlinearity, and a closed-loop self-oscillation circuit (e.g., a phase-locked loop) is employed to enable continuous frequency measurement [22].

When an external acceleration *a* is applied to the accelerometer, the amplified inertial force is applied axially to the resonators. Consequently, the equivalent stiffness of the resonators changes due to the axial force, expressed as shown below [23]

(1) $$k_{eff}=C_{1}\frac{EI}{L^{3}}+C_{2}\frac{F}{L}$$

where *C*_1_ and *C*_2_ are dimensionless constants determined by the mode shape function of the resonant beam, *E* is the Young’s modulus of silicon, *L* is the length of the resonant beam, *I* is the moment of inertia, and *F* is the axial force exerted on the resonant beam. For a beam with a rectangular cross-section of width *w* and thickness *t*, the moment of inertia is calculated as *I* = *wt*^3^/12. *F* is positive and negative for the beam under tension and compression, respectively. The resonant frequency of the resonators with the axial force is then given by

(2) $$f=\frac{1}{2\pi }\sqrt{\frac{k_{eff}}{m_{eff}}}=f_{0}\sqrt{1+F\frac{C_{2}L^{2}}{C_{1}EI}}$$

where *m_eff_* is the equivalent mass and *f*_0_ is the natural frequency of the resonators in the absence of the axial force, thus being independent of the external acceleration.

Equation (2) indicates that the resonant frequency depends on the axial force *F*. Because *F* is proportional to the external acceleration *a*, the acceleration can be determined by measuring the frequency shift.

## 3. Optimization Model of Microlevers Considering the Arm’s Shape

### 3.1. Objective Function

An optimization model for microlevers, aimed at improving the sensitivity of MEMS resonant accelerometers, is established through finite element simulation. The first step involves deriving the objective function.

**Figure 2:**
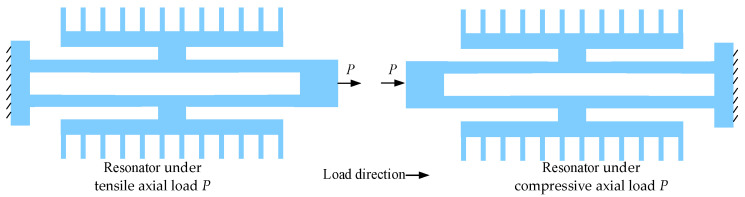
A schematic diagram of the resonators under axial loads. The axial loads are the amplified inertial force. Because the tuning fork resonators are symmetrically distributed with each other, one must be pulled, and the other must be compressed.

The sensitivity of the MEMS resonant accelerometer is defined as the natural frequency shift per unit of acceleration [24]:

(3) $$S=\frac{f_{t}-f_{c}}{a}$$

where *f_t_* and *f_c_* represent the natural frequencies of the resonators under tensile and compressive axial loads induced by the external acceleration, respectively (as shown in Figure 2). The dependencies of the natural frequencies *f_t_* and *f_c_* on the external acceleration are expressed as shown below [17,19]

(4) $$\begin{array}{l} f_{t}=f_{0}\left(1+\beta a\right)\\ f_{c}=f_{0}\left(1-\beta a\right) \end{array}$$

where *β* is a coefficient determined by the structure of the accelerometer. Substituting Equation (4) into Equation (3) yields the objective function, which is given by

(5) $$S=2\beta f_{0}=\frac{2\left(f_{t}-f_{0}\right)}{a}$$

As can be observed from Equation (5), the sensitivity increases with *f_t_*, since *f_0_* is only related to the structure of the resonator and is independent of the external acceleration. Thus, during optimization of the microlever, only the natural frequency of the resonator under tensile axial loads needs to be calculated via finite element simulation, thereby reducing the simulation time.

### 3.2. Optimization Variables and Constraints

**Figure 3:**
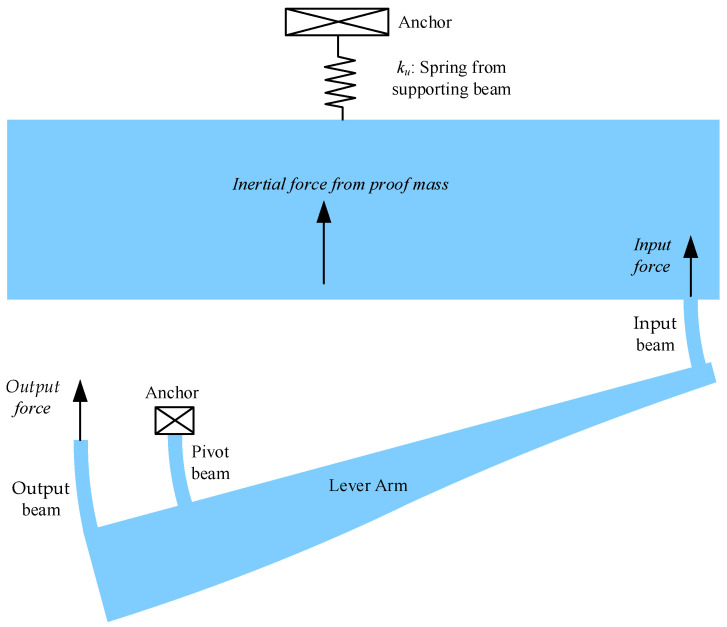
A schematic diagram of microlevers’ working principle. *k_u_* represents the equivalent stiffness of the four supported beams, which only allows vertical movement of the proof mass. The output force is input to tuning fork resonators to change the natural frequencies.

Fabricating rotational joints on a microscale is impractical, so compliant beams, including pivot beams, output beams, and input beams, are employed in the microlevers, as shown in Figure 3. In addition to improving the force amplification factor from the input force to the output force, the bending stiffness of the pivot beams, output beams, and input beams must also be matched to that of the supported beams. This ensures that most inertial forces are transmitted as the input force to the microlevers.

The bending stiffness of pivot beams, output beams, input beams, and supported beams can be reduced by increasing their length or decreasing their width. In this study, a fixed width of 6 μm is adopted for the pivot, output, and input beams to achieve low bending stiffness, and the length of the supported beams is fixed at 1160 μm. Consequently, the adjustable lengths of the pivot beams (*l_p_*), output beams (*l_o_*), and input beams (*l_i_*) and the width of the supported beams (*w_s_*) become the optimization variables.

Additionally, the force amplification factor depends on the pivot position, i.e., the distance from the pivot to the output beam *l_po_*, so *l_po_* is also an optimization variable.

**Figure 4:**
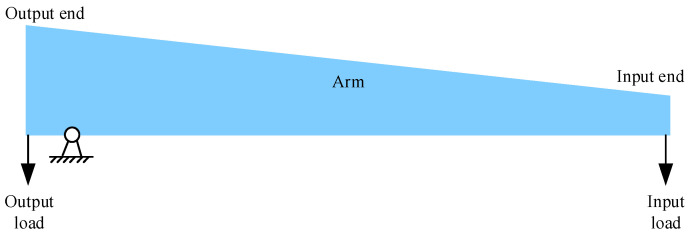
Load condition of the microlevers. The output load and pivot constrain the displacement of the arm’s left end, so the arm can be approximated as a cantilever.

Finally, the microlever arm must be sufficiently strong to form a lever system with a high amplification factor [16,25]. This paper describes microlevers with a trapezoidal arm, as shown in Figure 4. The width of the trapezoidal arm decreases gradually along its length from the output end to the input end. Because the pivot is located far from the input end, the displacement at the input end is much larger than that at the output end. In other words, the load condition of the microlever arm resembles that of a cantilever beam. According to structural mechanics principles, a trapezoidal cantilever beam is stiffer than a rectangular cantilever beam of the same area [26]. Thus, a trapezoidal microlever can achieve equivalent stiffness in a smaller area compared to a rectangular microlever. Consequently, a trapezoidal microlever allows for a larger proof mass within a given die area, resulting in higher accelerometer sensitivity. The trapezoidal arm is divided into four equal segments, and its hypotenuses are described using linear interpolation.

(6) $$\begin{array}{l} y=y_{l2}+\frac{y_{l1}-y_{l2}}{\Delta x}\left(x+2\Delta x\right);\left(-2\Delta x\leq x\leq -\Delta x\right)\\ y=y_{l1}-\frac{y_{l1}}{\Delta x}\left(x+\Delta x\right);\;\;\left(-\Delta x\leq x\leq 0\right)\\ y=\frac{y_{r1}}{\Delta x}x;\;\;\;\;\;\;\;\left(0\leq x\leq \Delta x\right)\\ y=y_{r1}+\frac{y_{r2}-y_{r1}}{\Delta x}\left(x-\Delta x\right);\left(\Delta x\leq x\leq 2\Delta x\right) \end{array}$$

**Figure 5:**
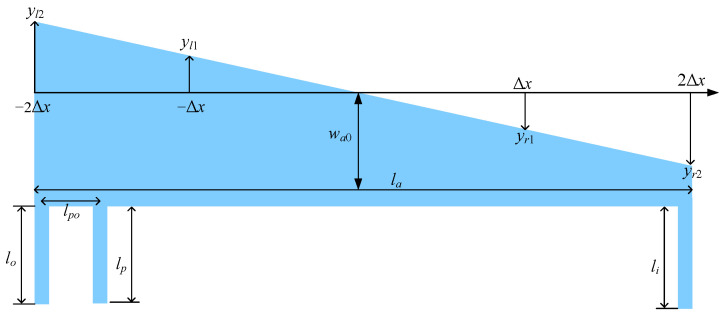
Geometric dimensions of a trapezoidal microlever. In addition to the dimensions of pivot beams, output beams, and input beams, the width of the arm must also be parameterized, because the shape of the arm is considered in the optimization in this work.

The shape of the trapezoidal arm is determined by the vertical coordinates of the hypotenuses (*y_l_*_1_, *y_l_*_2_, *y_r_*_1_, and *y_r_*_2_) and its middle width (*w_a_*_0_), as shown in Figure 5. Thus, the optimization variables include the aforementioned vertical coordinates and the middle width of the trapezoidal arm.

However, the vertical coordinates and the middle width must satisfy constraints related to the trapezoidal shape, as well as a minimum arm width specified by the fabrication process.

(7) $$\begin{array}{l} y_{l2}\geq y_{l1}\geq 0\\ y_{r2}\geq y_{r1}\geq 0\\ w_{0}-y_{r2}\geq w_{min} \end{array}$$

In summary, the optimization model for the trapezoidal microlevers can be expressed as

(8) $$\begin{array}{l} \max S=S\left(l_{p},l_{o},l_{i},l_{po},w_{s},w_{a0},y_{l1},y_{l2},y_{r1},y_{r2}\right)\\ \;\;l_{p\;min}\leq l_{p}\leq l_{p\;max};l_{o\;min}\leq l_{o}\leq l_{o\;max};l_{i\;min}\leq l_{i}\leq l_{i\;max};\\ \;\;l_{po\;min}\leq l_{po}\leq l_{po\;max};w_{s\;min}\leq w_{s}\leq w_{s\;max};w_{a0}-y_{r2}\geq w_{amin}\\ \;\;y_{l2}\geq y_{l1}\geq 0;y_{r2}\geq y_{r1}\geq 0 \end{array}$$

## 4. Shape Optimization Model of Resonators

**Figure 6:**
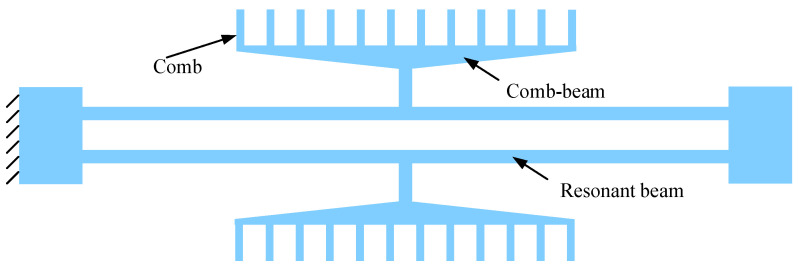
A schematic diagram of the resonator with rectangular resonant beams. In order to reduce the middle mass to enhance the sensitivity of the resonators, a trapezoidal comb beam is designed. The trapezoidal shape has lower mass compared to the rectangular one with the same stiffness.

**Figure 7:**
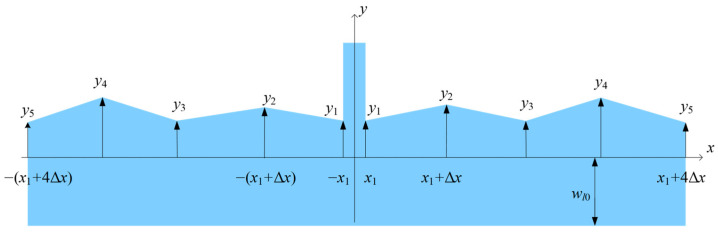
A schematic diagram of geometric dimensions of the non-rectangular resonant beam. The varying width is parameterized as the optimization variable. The shape of the resonant beam is symmetrical about the Y-axis.

Current resonant accelerometers typically use rectangular resonant beams, as shown in Figure 6. This study proposes using non-rectangular resonant beams to improve sensitivity, as shown in Figure 7, where the inner edge is curved. This non-rectangular beam is horizontally symmetrical to ensure that the comb actuators move vertically. A trapezoidal comb beam is also employed to reduce the middle mass, which further enhances the sensitivity of the resonators.

To increase the sensitivity while simultaneously keeping the fundamental frequency constant, a bi-objective function is proposed as follows:

(9) $$S_{y}=q_{1}\left|f_{0}-f_{0}^{\prime }\right|-q_{2}(f_{t}-f_{0})/P$$

where *q*_1_ and *q*_2_ denote the weight coefficients, which are introduced to ensure that the two terms are of the same order of magnitude. *f*_0_ and *f*_0_′ denote the unloaded natural frequencies of the resonant beams before and after optimization, respectively. *P* represents the external axial load applied to the resonant beams.

The curved edge is modeled as an even function to maintain symmetry. The resonator beam is divided into eight segments as shown in Figure 7, and, thus, the curved edge is described by a linear interpolation:

(10) $$\mathrm{If}\;x\;>\;0,\;\left\{\begin{array}{l} y\left(x\right)=y_{i-1}+\frac{y_{i}-y_{i-1}}{\Delta x}\left(x-\left[x_{1}+(i-2)\Delta x\right]\right)\\ (i-2)\Delta x+x_{1}\leq x\leq (i-1)\Delta x+x_{1} \end{array}\right.$$

(11) $$\mathrm{If}\;x<0,\;y\left(x\right)=y\left(-x\right)$$

where Δ*x* represents the length of each segment. The optimization model can be expressed as

(12) $$\begin{array}{l} \min S_{y}=S\left(w_{r0},y_{1},\cdots ,y_{5}\right)\\ \;\;y_{imin}\leq y_{i}\leq y_{imax}\\ where\;i=1,2,\cdots ,5 \end{array}$$

where *w_r_*_0_ is the basic width of the resonant beam.

## 5. Genetic Algorithm and Finite Element Model

To avoid local optima, the genetic algorithm was employed to search for the solution. On the platform of COMSOL Multiphysics 6.2 with MATLAB R2024b, the frequency was obtained by the finite element simulation based on COMSOL Multiphysics 6.2, and the optimization variables iteration was executed in MATLAB using the built-in *ga* function for the genetic algorithm. The parameter settings for the *ga* function are listed in Table 1, and the constraints subject to the lower and upper bounds are defined by the values listed in Table 2.

The parameters used for the optimization of the microlevers are listed in Table 2. As employed in prior studies [14], the width and length of the resonant beams are fixed at 8 μm and 1100 μm, respectively, which ensures that their natural frequency is approximately 20 kHz; this has been widely adopted in current studies to better match the parameters of the interface circuit [17,19,24,27,28]. A microlever arm with a length of 1472 μm was selected to achieve a high force amplification factor.

**Figure 8:**
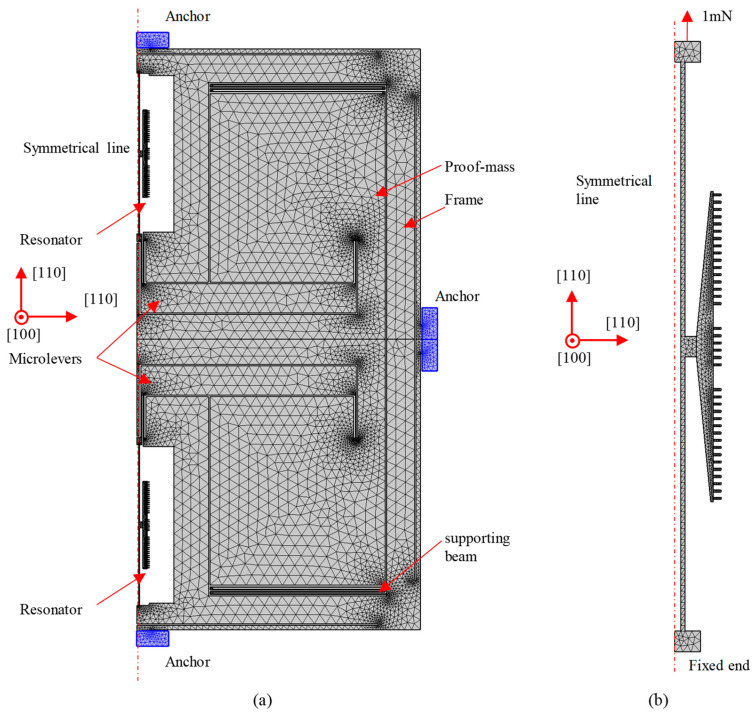
Details for the finite element models used in the optimization. (**a**) Model for the optimization of the microlevers; (**b**) model for the optimization of the resonators.

The 2D finite element models for the optimization of the microlevers and resonators are shown in Figure 8. In the model for the optimization of the microlevers, a frame is placed between the anchors and other elements, such as supported beams, resonators, and microlevers, to isolate residual stress. In the model for the optimization of the fork resonators, the bottom end is fixed, and the axial force is applied on the top end. In both models, the mesh size named extra fine in COMSOL Multiphysics 6.2 is used. The density and anisotropic elastic properties of single-crystal silicon in the finite element model are set using the data from the literature [29]. The [110] crystal orientations match the axial directions of resonators and supporting beams, and the [100] crystal orientation is vertical to the plane. In the finite element simulation, the solver tolerance is 0.001.

## 6. Optimization Results and Discussion

### 6.1. Optimization Results on Microlevers

**Figure 9:**
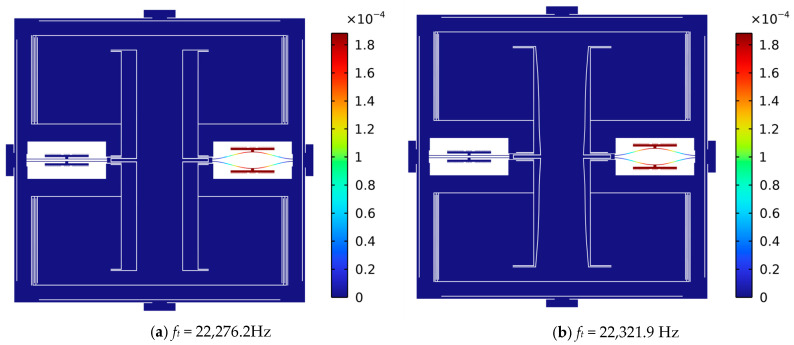
Simulated mode shape of the whole accelerometer under a load of 1g external acceleration. (**a**) Pre-optimization of microlever. (**b**) Post-optimization of microlever. Though the finite element simulation used a semi-model, the simulation results present the full mode shape realized by the symmetric operation.

The optimized results are listed in Table 3. The designed resonator exhibits a natural frequency close to 22,200 Hz. Natural frequencies in the vicinity of 20 kHz have been widely adopted in recent studies to better match the parameters of the interface circuit. Under an external acceleration of 1 g, the inertial force induced tensile stress in the lower resonator, leading to a significant increase in the natural frequency of the lower resonator. Using the simulated natural frequency results, the sensitivity was calculated and is listed in Table 3. After optimizing the microlevers, the sensitivity was as high as 286.9 Hz/g, which is much higher than the pre-optimization result with a sensitivity of 193.5 Hz/g. A 48.3% increase in sensitivity indicates that the optimization of the microlevers significantly improved the sensitivity of the accelerometer, see Figure 9.

### 6.2. Optimization Results on Resonators

**Figure 10:**
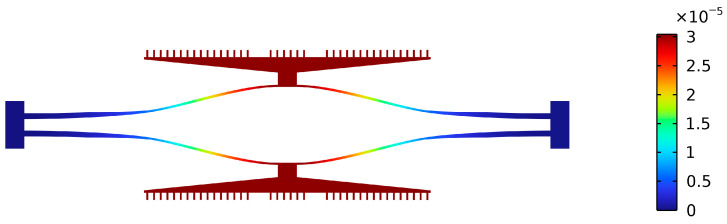
Simulated mode shape of the resonators under a load of 1 mN. Although the resonant beam is no longer rectangular, the modal shape still only allows for vertical movement of the comb teeth.

The parameters used for the optimization of the resonator are listed in Table 4, and the resonator itself is shown in Figure 10. It can be seen that the resonant beam is no longer rectangular. However, the modal shape still only allows for vertical movement of the comb teeth, which ensures the characteristics of the capacitor remain linear. The sensitivity of the resonator is increased from 727 Hz/mN to 1338.5 Hz/mN, representing an improvement of 84.1%, while the fundamental frequency remains constant, see Table 5.

### 6.3. Overall Optimization Results

**Figure 11:**
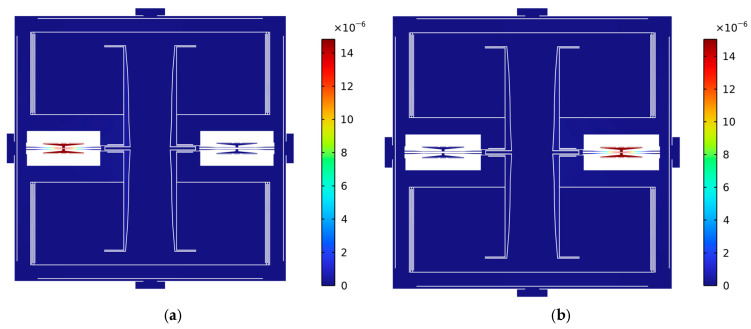
The simulated mode shape of the whole accelerometer after integrating the optimized microlever and resonator configurations, (**a**) Mode of the lower resonator, 22,706.4 Hz. (**a**) Mode of the upper resonator, 22,226.2 Hz. The external acceleration is 1 g.

Integrating the optimized microlever and resonator configurations yields the final mode shapes of the accelerometer, as shown in Figure 11. This integration results in a very high sensitivity of 480.2 Hz/g, representing a 148.2% improvement compared to the sensitivity before optimization. This significant improvement verifies the effectiveness of shape optimization for microlevers and resonators.

Simulations considering fabrication errors were conducted to investigate robustness. Fabrication errors were applied to the widths of the supporting beams, pivot beams, output beams, input beams, and resonant beams, which are sensitive to fabrication uncertainty. The results listed in Table 6 show that the sensitivity remains very high, and the fundamental frequency stays close to 20 kHz even in the presence of manufacturing errors. Consequently, the accelerometer design exhibits good robustness.

A comparison between this work and previously reported studies is presented in Table 7. The size of the proof mass also affects sensitivity, and previously reported studies have typically employed different proof mass sizes. Therefore, a parameter defined as the sensitivity per proof mass is used to compare the sensitivities achieved in different studies. The sensitivity per proof mass achieved in this work is higher than that reported in previous studies employing mechanical stiffness tuning. In summary, shape optimization provides a high-sensitivity solution for MEMS accelerometers in compact spaces, promoting performance advancement.

**Figure 12:**
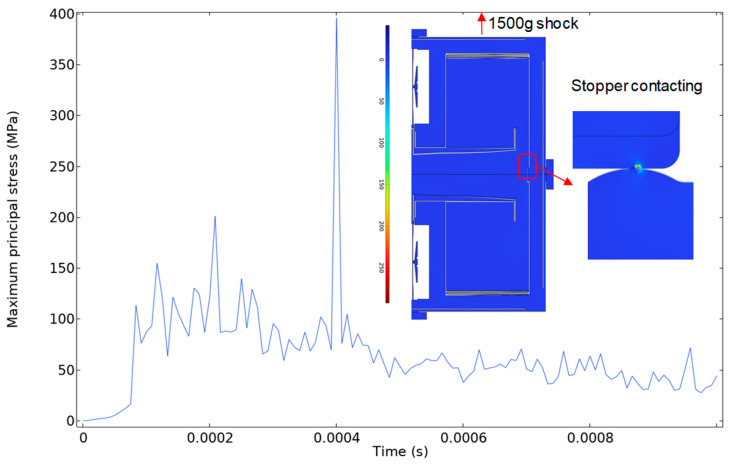
The simulation result of the final optimized accelerometer under the shock load of half-sine 1500 g with a 0.5 ms duration. The internal image represents the 1st principal stress distribution at time of 0.0004 s.

In order to verify the shock performance, we conducted a finite element simulation of the final optimized accelerometer under a shock load of half-sine 1500 g with a 0.5 ms duration. This level of shock load is the requirement of automotive standard. A small stopper with a 7 μm gap was created between the proof mass and the frame. The simulation result shown in Figure 12 indicates that the maximum first principal stress was approximately 400 MPa, which is lower than the tensile strength of the brittle monocrystalline silicon, 600 MPa [30]. As a result, even under high shock loads at the vehicle specification level, the final optimized accelerometer is still safe.

**Figure 13:**
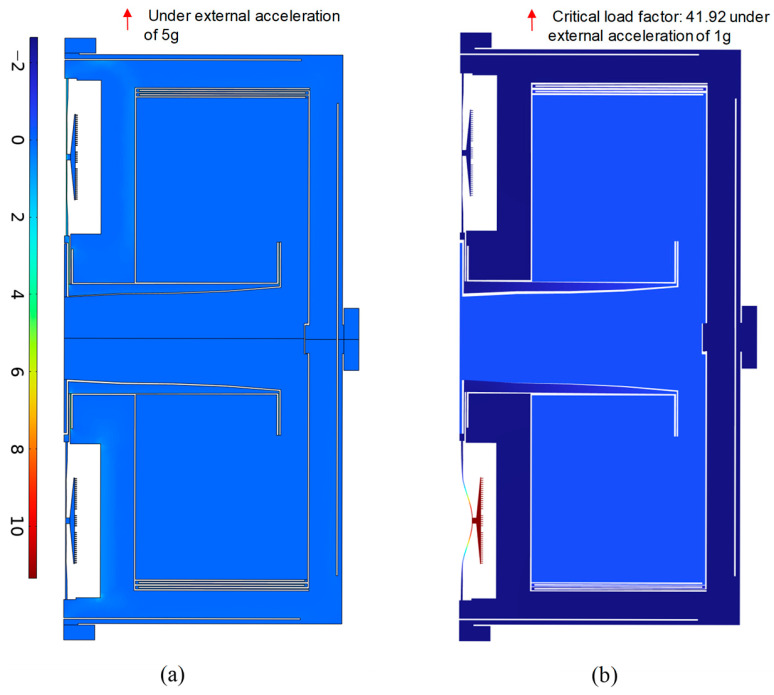
The simulation result of maximum stress and buckling for the final optimized accelerometer. (**a**) First principal stress distribution at the peak load of 5 g; (**b**) buckling analysis under a load of 1 g, resulting in a critical load factor of up to 41.92.

The finite element simulation of maximum stress under the peak load of 5 g was implemented for the final optimized accelerometer, and the result is shown in Figure 13a. The maximum first principal stress was much lower than the tensile strength of the brittle monocrystalline silicon. On the other hand, the buckling analysis under a load of 1 g showed that the critical load factor was as high as 41.92, i.e., the buckling of the final optimized accelerometer occurred when the load was higher than 41.92 g, as shown in Figure 13b. In summary, under the peak load of 5 g, the final optimized accelerometer is safe.

**Table 7:** A comparison of this work with previously reported works. MST represents the mechanical stiffness tuning.

Authors	Sensitivity (Hz/g)	Proof Mass (Microgram)	Sensitivity per Proof Mass (Hz/g/Microgram)	Principle
Yin et al. [19]	244.15	2104	0.116	MST
Park et al. [31]	188	872	0.216	MST
Zhao et al. [32]	140	1571	0.089	MST
Gao et al. [24]	200	4543	0.044	MST
Huang et al. [16]	59.12	499	0.118	MST
Zhang et al. [18]	211.5	1700	0.144	MST
Zhang et al. [28]	31.56	309	0.102	MST
Xia et al. [33]	108.72 (*x*-axis) 106.89 (*y*-axis)	9758	0.011 (*x*-axis) 0.011 (*y*-axis)	MST
Huang et al. [34]	64.57	1140	0.0566	MST
Cai et al. [35]	512	1500	0.34	MST
This work	480.2	1178	0.408	MST

## 7. Conclusions

This study proposes shape-based optimization for microlevers and resonators to improve the sensitivity of MEMS resonant accelerometers.

The optimized trapezoidal microlevers achieve stiffness equivalent to that of rectangular microlevers while occupying a smaller area, allowing for an increase in the proof mass area to achieve higher sensitivity. After optimization, the sensitivity increases to 286.9 Hz/g. Non-rectangular resonators are proposed and optimized to make the frequency more sensitive to axial force. The shape optimization of the non-rectangular resonators increases the sensitivity to axial force from 727 Hz/mN to 1338.5 Hz/mN.

Finally, the optimized microlevers and resonant beams are integrated into an accelerometer. Compared with the pre-optimization design, the sensitivity increases from 193.5 Hz/g to 480.2 Hz/g, representing a 148.2% improvement. A comparison with previous studies shows that this design achieves a higher sensitivity per proof mass area. Thus, optimizing trapezoidal microlevers and resonant beams enables the development of high-sensitivity MEMS resonant accelerometers within compact spaces.

Future work may involve experiments to verify the sensitivity optimization achieved by considering the shapes of microlevers and resonant beams. It is also valuable to optimize other performances, such as scale-factor linearity, bias stability, and noise density.

## Figures and Tables

**Table 1:** Parameter settings and description for the *ga* function implementing the genetic algorithm.

Parameters	Optimization for Microlevers	Optimization for Resonators	Description
PopulationSize	200	50	Size of the population
EliteCount	10	3	Specifying how many individuals survived to the next generation
SelectionFcn	selectionstochunif	selectionstochunif	Function that selects parents of crossover and mutated children
CrossoverFraction	0.8	0.8	The fraction of the population in the next generation
MutationFcn	mutationgaussian	mutationgaussian	Function that produces mutated children
FunctionTolerance	1%	1%	The relative change in function value for the termination criteria
MaxGenerations	50	50	Maximum number of iterations before the algorithm halts

**Table 2:** Parameters and bounds on microlevers.

Parameters	Value	Unit
Thickness of device layer(*t*)	60	μm
Width of the resonator (*w_r_*)	8	μm
Length of resonator (*l_r_*)	1100	μm
Width of pivot, output and input beam (*w_p_*, *w_o_*, *w_i_*)	6	μm
Arm length (*l_a_*)	1472	μm
Lower and upper bounds of pivot length (*l_pmin_*; *l_pmax_*)	50; 300	μm
Lower and upper bounds of output length (*l_omin_*; *l_omax_*)	50; 300	μm
Lower and upper bounds of input length (*l_imin_*; *l_imax_*)	50; 300	μm
Lower and upper bounds of the distance from pivot beam to output beam (*l_opmin_*; *l_opmax_*)	6; 13	μm
Lower and upper bounds of arm middle width (*w_0min_*; *w_0max_*)	50; 200	μm
Lower and upper bounds of vertical coordinates of arm’s curved edge (*y_kmin_*; *y_kmax_*)	0; 50	μm

**Table 3:** Sensitivity after the optimization of the microlever. Pre-optimization sensitivity is listed for comparison.

Pre or Post	Optimized Variables (μm)	Sensitivity (Hz/g)
Pre-optimization	*l_p_* = 150 *l_o_* = 150, *l_i_* = 150.5, *l_po_* = 12, *w_s_* = 10, *w_a_*_0_ = 200, *y_l_*_1_ = 0, *y_l_*_2_ = 0, *y_r_*_1_ = 0, *y_r_*_2_ = 0	193.5
Post-optimization	*l_p_* = 246 *l_o_* = 288, *l_i_* = 292.5, *l_po_* = 8.35, *w_s_* = 6.08, *w_a_*_0_ = 60, *y_l_*_1_ = 4.5, *y_l_*_2_ = 23.5, *y_r_*_1_ = −14.5, *y_r_*_2_ = −44	286.9

**Table 4:** Parameters of the optimization model for resonators.

Parameters	Value	Unit
Base width of the resonant beam (*w_r_*_0_)	4	μm
Weight coefficient (*q*_1_)	0.5	1/mN
Weight coefficient (*q*_2_)	1	1
Load (*P*)	1	mN

**Table 5:** Sensitivity after the optimization of the resonators. Pre-optimization sensitivity is listed for comparison.

Pre or Post	Optimized Variables (μm)	Fundamental Frequency Without External Acceleration (Hz)	Sensitivity to Load (Hz/mN)
Pre-optimization	*y*_1_ = 4.00, *y*_2_ = 4.00, *y*_3_ = 4.00, *y*_4_ = 4.00, *y*_5_ = 4.00	22,187.0	727.0
Post-optimization	*y*_1_ = 0.18, *y*_2_ = 1.47, *y*_3_ = 0.20, *y*_4_ =7.19, *y*_5_ =8.25	22,188.9	1338.5

**Table 6:** Dependence of sensitivity and fundamental frequency on fabrication error.

Fabrication Error (μm)	Sensitivity (Hz/g)	Fundamental Frequency Without External Acceleration (Hz)
−0.2	534.7	21,257.0
0.2	430.7	23,602.7

## Data Availability

Data will be made available upon request through personal contact with the corresponding author at the following email address: hejiangbo@foxmail.com.
